# Promoter Methylation in Head and Neck Squamous Cell Carcinoma Cell Lines Is Significantly Different than Methylation in Primary Tumors and Xenografts

**DOI:** 10.1371/journal.pone.0020584

**Published:** 2011-05-26

**Authors:** Patrick T. Hennessey, Michael F. Ochs, Wojciech W. Mydlarz, Wayne Hsueh, Leslie Cope, Wayne Yu, Joseph A. Califano

**Affiliations:** 1 Department of Otolaryngology-Head and Neck Surgery, Johns Hopkins Medical Institutions, Baltimore, Maryland, United States of America; 2 Department of Oncology, Johns Hopkins Medical Institutions, Baltimore, Maryland, United States of America; 3 Division of Biostatistics and Bioinformatics, Johns Hopkins Medical Institutions, Baltimore, Maryland, United States of America; 4 Feinberg School of Medicine, Northwestern University, Chicago, Illinois, United States of America; 5 Sidney Kimmel Comprehensive Cancer Center, Johns Hopkins Medical Institutions, Baltimore, Maryland, United States of America; 6 Milton J. Dance Head and Neck Center, Greater Baltimore Medical Center, Baltimore, Maryland, United States of America; National Cancer Institute, United States of America

## Abstract

Studies designed to identify novel methylation events related to cancer often employ cancer cell lines in the discovery phase of the experiments and have a relatively low rate of discovery of cancer-related methylation events. An alternative algorithm for discovery of novel methylation in cancer uses primary tumor-derived xenografts instead of cell lines as the primary source of nucleic acid for evaluation. We evaluated DNA extracted from primary head and neck squamous cell carcinomas (HNSCC), xenografts grown from these primary tumors in nude mice, HNSCC-derived cell lines, normal oral mucosal samples, and minimally transformed oral keratinocyte-derived cell lines using Illumina Infinum Humanmethylation 27 genome-wide methylation microarrays. We found >2,200 statistically significant methylation differences between cancer cell lines and primary tumors and when comparing normal oral mucosa to keratinocyte cell lines. We found no statistically significant promoter methylation differences between primary tumor xenografts and primary tumors. This study demonstrates that tumor-derived xenografts are highly accurate representations of promoter methylation in primary tumors and that cancer derived cell lines have significant drawbacks for discovery of promoter methylation alterations in primary tumors. These findings also support use of primary tumor xenografts for the study of methylation in cancer, drug discovery, and the development of personalized cancer treatments.

## Introduction

It is well established that alterations in promoter methylation and the resulting changes in gene expression play a critical role in the pathogenesis of many human cancers [1]. Hypermethylation of CpG islands in promoter regions is associated with transcriptional repression of tumor suppressor genes [2], [3], while hypomethylation is associated with activation of oncogenes [3]. A common algorithm used for the identification of novel aberrant methylation events involves applying pharmacologic demethylating agents, such as 5-aza-2′-deoxycytidine (5-aza-dC) to cancer cell lines, assaying the treated cells for altered gene expression, and then validating the methylation status of the differentially expressed genes in primary tumors and normal tissue [4], [5], [6]. This algorithm, however, often results in a low yield of cancer-specific methylation of genes [4], [6]. Although cancer cell lines are attractive for studying methylation in cancer it has been established that cancer cell line DNA is hypermethylated compared to primary tissue, and it is suggested that cell lines do not faithfully represent the methylation status of primary tumors [7], [8], [9], [10], [11], possibly due alterations in methylation that allow cells to survive in culture [12]. Unlike cancer cell lines, tumor xenografts are grown *in vivo* in mice and are not subjected to frequent high serum environments and frequent passages which have been implcated in resulting altered methylation [12]. To date, however, no evaluation has been conducted to compare they genome-wide methylation profile of tumor xenografts and cancer cell lines to determine which of these tissues best correspond to primary tumors. In this study we used head and neck squamous cell carcinomas (HNSCCs) as a model system to investigate the methylation profiles of primary tumors, tumor xenografts (xenografts), normal mucosa, cancer cell lines and normal oral keratinocyte-derived cell lines using genome-wide methylation profiling microarrays to determine whether cell lines or tumor xenografts better represent the methylation profile of primary tumors.

## Methods

### Human tissue samples

All human HNSCC tissue samples and normal mucosal tissues were obtained and used according to the policies of the Johns Hopkins Medical Institutions and Greater Baltimore Medical Center institutional review boards. Surgical specimens were obtained from patients who underwent surgery at John Hopkins Hospital or Greater Baltimore Medical Center, and the collection of these tissue specimens was approved by these institutional review boards. Written informed consent for participation in this study was obtained from all patients prior to surgery. A small portion of the tissue removed during the procedure was processed for the study after routine pathological analysis per the routine standard care for the patients. After review by a pathologist, a piece of each tumor specimen was de-identified from the patient and given a unique identification number and was immediately taken, without freezing, to be implanted in nude mice for xenograft generation. A separate piece of the same tumor was microdissected by a pathologist to assure that greater than 80% of tissue contained HNSCC prior to being snap frozen in liquid nitrogen. Three primary tumor specimens are included in this study. Seven normal tissue specimens were obtained from patients who underwent uvulopalatopharyngoplasty (UPPP) for sleep apnea. After review by a pathologist, a section of dissected mucosal layer from discarded UPPP specimens was immediately frozen in liquid nitrogen. All specimens were stored at −80°C until processing.

### Xenograft Generation

Our protocol for xenograft implantation was approved and carried out in strict adherence to the policies and guidelines set forth by The Johns Hopkins University Animal Care and Use Committee (Protocol #: MO08M248). All surgery was performed under isoflurane-induced anesthesia and every effort was made to minimize suffering. Briefly, fresh tissue from 3 primary HNSCCs was implanted in the flank of nude mice at the Johns Hopkins Hospital. Prior to implantation the xenograft area was sterilized using iodine solution. Two small 2 mm incisions were made on each side of the flanks using sterile scissors, and tumor tissue measuring 2 mm×2 mm and treated with Matrigel were implanted under the skin. Tumors were allowed to grow until tumor volume equaled 20 mm×20 mm. Animals were then euthanized by carbon dioxide asphyxiation followed by cervical dislocation, as approved by the AVMA panel on Euthanasia, and the xenografts were harvested.

### Cell Lines and Culturing Conditions

JHU-O11 and JHU-O22 cell lines were created from primary HNSCCs in the Division of Head and Neck Cancer Research, the Johns Hopkins University (Baltimore, MD).[13] UM22A and UM22B cell lines were provided by Ajay Verma (Merck & Co., North Wales, PA) and FaDu cells were obtained from the American Type Culture Collection. OKF6 cells are a minimally transformed oral keratinocyte line donated by Dr. James Rheinwald (Department of Dermatology, Brigham and Women's Hospital and Harvard Skin Disease Research Center). NOK-SI cells are normal oral keratinocytes that spontaneously immortalized and were provided by Dr. Silvio Gutkind (National Institutes of Health, Bethesda, MD).

JHU-O11, JHU-O22 and FaDu cell lines were cultured in RPMI-1640 media supplemented with 10% FBS and 1% penicillin-streptomycin. UM22A and UM22B cell lines were cultured in high-glucose DMEM with 10% FBS and 1% penicillin-streptomycin. OKF6 cell lines were grown in keratinocyte serum-free medium supplemented with bovine pituitary extract (25 µg/ml), calcium chloride (0.4 mM), epidermal growth factor (0.2 ng/ml) and 1% penicillin-streptomycin that was filtered through 0.2-µm pore-size sterilization filter prior to use. NOK-SI cells were grown in fully supplemented keratinocyte serum-free medium. All media components were obtained from Gibco Invitrogen Corporation (Carlsbad, CA). Cell growth conditions were maintained at 37°C in an atmosphere of 5% carbon dioxide and 95% relative humidity.

### DNA extraction

DNA was extracted from all samples by digestion with 50 µg/mL proteinase K (Boehringer, Mannheim, Germany) in the presence of 1% SDS at 48°C overnight, followed by phenol/chloroform extraction and ethanol precipitation.

### Bisulfite conversion for microarrays

Bisulfite conversion of genomic DNA was done with the EZ DNA methylation Kit (Zymo Research, D5002) by following manufacturer's protocol with modifications for Illumina Infinium Methylation Assay in the Johns Hopkins microarray core.

### Microarray analysis

Bisulfite-converted genomic DNA was analyzed using Illumina's Infinium Human Methylation27 Beadchip Kit (WG-311-1202) in the Johns Hopkins microarray core. Beadchip contains 27,578 CpG loci covering more than 14,000 human RefSeq genes at single-nucleotide resolution. Chip process and data analysis were performed by using reagents provided in the kit and following manufacturer's manual. Data were extracted and summarized using BeadStudio v3.0 software. Arrays that did not pass quality control in terms of β-distributions and expected p-values across the arrays were removed from further analysis. All microarray data are MIAME compliant and have been submitted to the Gene Expression Omnibus (GEO Accession ID: GSE24787).

### Comparison between specimen groups

Data were preprocessed using a custom R script to retain only methylation probes containing ≥3 CpG sites per probe. These probes were chosen based on our prior experience that probes that included ≥3 CpG sites per probe demonstrated consistently reproducible methylation status by bisulfite sequencing, providing a higher quality read. There were 12,023 probes retained for 3 tumors, 3 xenografts, 7 UPPPs, 5 cancer cell lines, and 2 normal mucosa-derived cell lines. Empirical Bayes comparisons were made between different tissues and cell lines using the limma package [14] in R/Bioconductor [15] to determine the difference in methylation between these groups.

### Unsupervised hierarchical clustering

Normalized data for the percentage methylated CpG sites for all high quality probes on the Illumina array were subjected to unsupervised hierarchical clustering analysis with Euclidean distance and average linkage using the MultiExperiment Viewer (MeV) application [16].

### Bisulfite conversion for sequencing

Bisulfite conversion of 2 ug genomic DNA from all samples was conducted using the Qiagen EpiTect bisulfite conversion kit per the manufacturers protocol. (Qiagen, Valencia, CA).

### Bisulfite sequencing

Bisulfite sequencing was conducted for the 3 CpG sites probed in the promoter regions of TM4SF19 (NCBI Accession #: NM_[13]8461.1; Chromosome 3:196,015,593–196,115,592 Illuminia probe ID: cg05445326;) and SERPINA12 (NCBI Accession #: NM_173850.2; Chromosome 14: 94,933,839–95,033,838; Illumina probe ID: cg05485062) (Table 1) as well as for four other probes on the array (Table S1). Probes to be included were chosen from a list of genes with significant differential methylation between tumor-xenografts and cell lines (Table S2). Probe location on the human genome was determined using the NCBI Basic Local Alignment Search Tool and the UCSC Genome Browser utilizing the March 2006 human reference assembly (NCBI Build 36.1). Bisulfite-treated DNA was amplified using primers designed using MethPrimer [17] to span the sequence of the probes used on the Illumina Humanmethylation27 array. Array sequence data were obtained from the “Illumina Human Methylation Sequence Data.csv” file available for download at www.illumina.com. Primer extension sequencing was performed by GENEWIZ, Inc (South Plainfield, NJ) using Applied Biosystems BigDye version 3.1. The reactions were then run on Applied Biosystem's 3730xl DNA Analyzer.

**Table 1:** Bisulfite sequencing primers use for validation of differentially methylated targets identified with the Illumina Humanmethylation27 microarrays.

Gene Name	Gene ID	Illumina Array ID	Forward Primer	Reverse Primer
ZFN671	NM_024833	cg19246110	TTTTGTGTTGATGAGAATTTTGTTT	TACATACCCAATAAAAACCCAAAAA
TRIM58	NM_015431	cg07533148	ATAGTTTTTGTTTTAGGTGTATTTT	ATAAACTAAACCACACAACCCTCC
MAGEA3	NM_005362	cg07545232	TGAGGTTTTTTGTTTGAGGTGA	CATCAACTTCAAAACCCTAAAAAATA
TM4SF19	NM_138461	cg05445326	TAGGATTTTTTTTAGGAGGGTTAGG	TAATACAAAAACCATACAACACATC
SERPINA12	NM_173850	cg05485062	GTAGGGAGTATAGTGGAGGGTTTTAA	AAATACAAACATCCCCAAATATCAA
IRF8	NM_002163	cg24826867	TTTTGGATTTTAGGTGTGAGGAG	TACCAATCTTTAAAAACAAACAAAC

*Gene name, NCBI Gene ID, Illumina Humanmethylation27 Array Probe ID and primer sequences are provided.

### Comparison of methylation array and bisulfite sequencing results

Methylation array data and bisulfite sequencing results were compared using the Freeman-Halton extension of the Fisher exact probability test for a three-rows by three-columns contingency table comparing methylation status. Bisulfite sequencing were read as fully methylated, partially methylated, and methylated based on analysis of sequencing tracings. If there was only a peak for a “C” or a “T” at the CpG site being analyzed the CpG site was called either methylated or unmethylated, respectively. If a peak had a mixed signal with both “C” and “T” peaks, the CpG site was deemed to be hemimethylated if the smaller peak was at least 20%–80% of the height of the larger peak. If the smaller peak was <20% the height of the larger peak the CpG site was called as either methylated or unmethylated based on the dominant peak. Illumlumina array data was read as fully methylated for β>0.75, hemimethylated for 0.25<β<0.75, and unmethylated for β<0.25. All p-values were corrected by the Bonferroni method for multiple testing.

### Gene Set Analysis

Gene set analysis (GSA) was performed comparing all tissue groupings. Results were sorted by t-statistic and a Wilcoxon rank sum test performed on sets of genes defined by the Broad Institute. The set used was the Gene Ontology Biological Process c5.bp.v2.5. This permitted GSA to be performed on all methylation probes passing the quality control. Resulting p-values from the Wilcoxon rank sum test were corrected with Benjamini-Hochberg multiple testing correction. An alpha value of 0.05 on the corrected p-value was used for significance.

## Results

Preliminary analysis was conducted using the 12,023 probes on the arrays that included ≥3 CpG islands per probe using empirical Bayes comparisons for all tissue types. This analysis revealed >2200 differentially methylation probes when comparing primary tissue (tumors or normal mucosa) to cell lines. There were 412 probes with significant differential methylation when comparing minimally transformed keratinocyte cell lines to HNSCC-derived cell lines, and 98 probes with a statistically significant difference in methylation between primary tumors and UPPPs. There were no statistically significant differences in methylation between tumors and primary tumor xenografts. (Table 2 and Tables S1, S2, S3, S4, and S5).

**Table 2:** Analysis of the 12,023 probes with ≥3 CpG sites/probe.

Comparison	Number of Genes with Significantly Different Methylation
Normal Tissue vs Cancer Cell Lines	2734
Normal Tissue vs Minimally Transformed Cell Lines	2727
Primary Tumors vs Cancer Cell Lines	2211
Minimally Transformed Cell Lines vs Cancer Cell Lines	412
Primary Tumor vs Normal Tissue	98
Primary Tumor vs Tumor-Derived Xenograft	0

*Analysis of the 12,023 genes with ≥3 CpG sites/probe. All samples were compared in a pairwise fashion. For all primary tissue compared to cell lines there were >2200 genes with a statistically significant methylation differences as determined by having a Benjamini-Hochberg adjusted p-value<0.05. When primary tumor was compared to normal tissue, 98 genes had a statistically significant difference in methylation. There were no statistically significant differences in methylation between tumors and tumor-derived xenografts.

**Figure 1:**
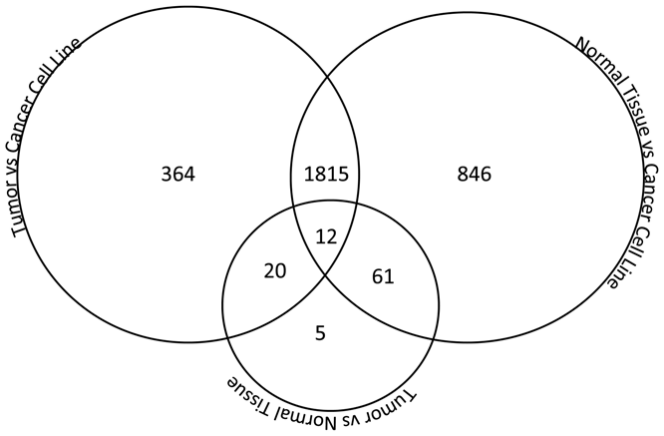
Venn Diagram of Methylation Differences Between Groups. There is a high degree of overlap between the probes differentially methylated in primary tumors vs. cancer cell lines, and in normal mucosa vs. cancer cell lines.

The individual statistically significantly differentially methylated probes from each comparison were then plotted on a Venn diagram to show the overlap between differentially methylated probes for all comparisons (Figure 1). There was a high degree of overlap between the genes that were differentially methylated in the tumor vs. cancer cell line comparison and the normal mucosa vs. cancer cell line comparison with 82% and 62% of the genes appearing in each group, respectively. Gene set analysis demonstrated differences in genes involved in cell cycle progression and cell growth (Table S6).

**Figure 2:**
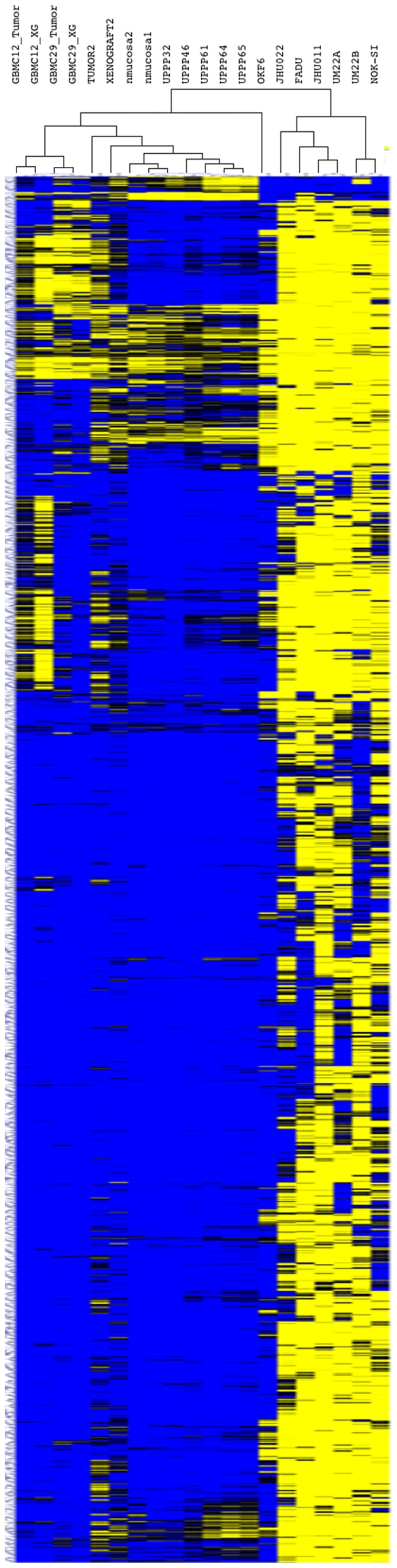
Unsupervised hierarchical clustering of methylation array data. All cell lines segregate from primary tissue by the second branch point of the dendrogram. All tumor/xenografts pairs cluster separately from the normal mucosa specimens by the fourth branch point of the dendrogram. Primary tumors and their corresponding xenografts cluster together after the terminal branch points.

The data from all 12,023 high quality probes (≥3 CpG islands) for each sample were then subjected to unsupervised hierarchical clustering analysis (Figure 2). After the first branch point on the dendrogram, 5 of the 6 cell lines segregated from all of the primary tissue specimens and one of the normal oral mucosa-derived cell lines (OKF-6). At the second branch point all of the tissues segregated from the OKF6 cells. At the terminal branches of the dendrogram all of the primary tumors segregated with their corresponding xenografts and all of the UPPPs segregated together. This demonstrates that methylation differences segregated primarily according to whether or not cell grew in adherent cell culture conditions, and that primary tumor xenografts most closely resemble primary tumors in promoter methylation patterns.

**Figure 3:**
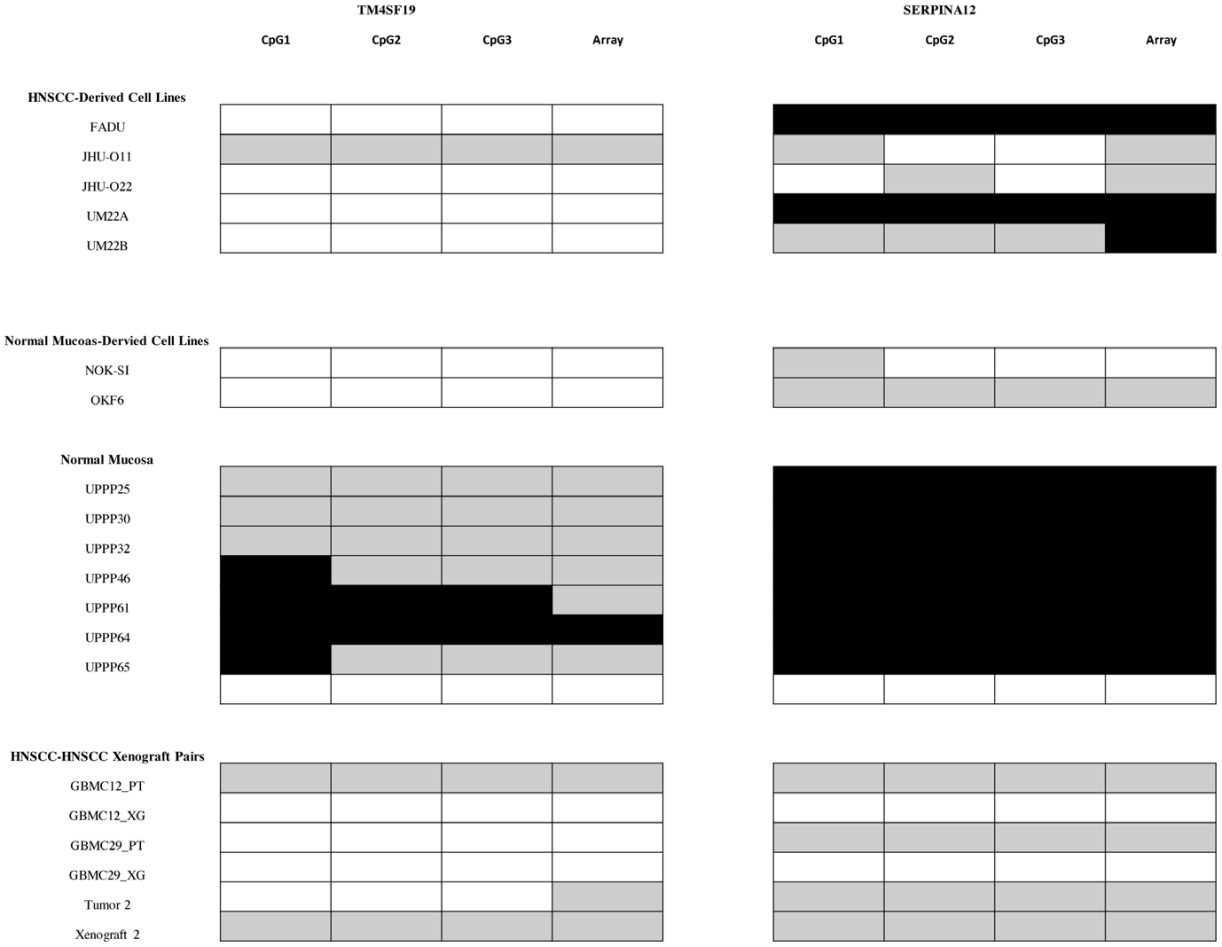
Representative bisulfite sequencing results for validation of microarray data. Bisulfite sequencing of the CpG sites probed by the array shows a high degree of concordance with the methylation array results. The methylation status of each CpG site is shown by bisulfite sequencing. Array intensity is an aggregate of the methylation status of the 3 CpG sites interrogated by each probe. All comparisons were significant after Bonferroni correction with a maximum corrected p-value of 2×10^−5^. Each CpG interrogated by bisulfite sequencing is included in the figure. Array signal is a summation of the methylation status of the CpG's being interrogated. (Black box = Full Methylation; Grey Box = Hemi-methylation; White box = Hypomethylation).

Bisulfite sequencing of the specific CpG sites probed by the Illumina arrays was conducted to validate the data obtained from the methylation microarrays. Sequencing data were obtained for six genes with high statistically significant differential methylation between tumors and cell lines for all specimens (Figure 3 and Table S7). The results of the methylation arrays and the bisulfite sequencing tests were compared using 3×3 contingency tables and the generalized Fisher test. The methylation arrays were ranked as unmethylated (beta<0.25), partially methylated (0.25<beta<0.75), and methylated (beta<0.75). All comparisons were significant after Bonferroni correction with a maximum corrected p-value of 2×10^−5^.

## Discussion

This is the first study to directly compare the whole-genome locus specific promoter methylation profiles of primary HNSCC, HNSCC tumor xenografts, and HNSCC cell lines. Although we had a relatively small sample size, our findings are consistent with several previous studies have compared methylation levels in tumors and cell lines from multiple different tissue types and have reported increased levels of methylation in tumor-derived cell lines when compared to primary tumors. [7], [10], [18], [19], including one study that compared tumor, xenograft, cell line and normal tissue whole-genome methylation profiles [20]. One recent study by Houshdaran and colleagues compared methylation profiles of ovarian epithelial cell carcinomas and cell lines at 1,505 GpG sites, representing 808 genes [21]. This study demonstrated a distinct difference in methhylation profiles of tumors and cell lines. 8% of the genes that we found to be differentially methylated in our study were also found to be differentially methylated in the smilar comparison made by Houshadaran and colleagues with ovarian cancers and ovarian cancer cell lines (Table S8). Another recent study by Milne and colleagues compared methylation profiles of a gastric cancer specimen, a xenograft from that cancer and 2 gastric cancer cell lines [22]. Similarly to our study, Milne and colleagues found that the methylation pattern in cell lines is different from primary tissues. This study, however, only looked at 38 genes. Paz and colleagues compared methylation of 15 genes in primary tumors and cell lines for 12 different tumor types, including HNSCC, and found that the methylation profile for these 15 genes is similar in primary tumors and corresponding cell lines [19]. This study, however, looked at methylation only for a subset of genes and did not evaluate the whole-genome promoter methylation profile. Of the 15 genes evaluated by Paz and colleagues, only DCC is seen in common with the 98 genes differentially methylated between normal tissue and primary tumors (Table S5, [19]).

The high degree of similarity between primary tumors and xenografts derived from those tumors, and the substantial discordance in methylation between primary tumors and cancer cell lines demonstrates that xenografts are more accurate models for promoter methylation in cancer than cancer cell lines. Although it is expected that the methylation of each individual xenograft would be similar to its corresponding primary tumor, all of the tumor-xenograft pairs had overall similar methylation patters as seen on hierarchical clustering while all of the cell lines had similar methylation patterns (Figure 2). It is possible that the different origin of the tumor samples and the cell line samples could explain some of the difference in their overall pattern of methylation, however, this is unlikely given the overall high-degree of similarity between the methylation pattern seen in the cancer cell lines, regardless of tissue of origin. A more likely explanation is that cell line methylation is affected by the tissue culture environment, which is not present in first generation xenografts. An important implication of our finding that xenografts have identical methylation to the primary tumor from which they were derived, is that researchers can use xenografts as a primary tissue source for studying methylation in cancer. By growing xenografts, the quantity of primary tissue available for study can be greatly expanded, allowing for more experiments to be conducted on each sample without a loss in the fidelity of the methylation status of the xenografts. Additionally, although discovery of promoter methylation alterations in cancer has classically been done using cell lines [4], [5], [6], it is possible to conduct demethylation *in vivo* in nude mice carrying tumor xenografts, by exposing the mice to systemic 5-aza-dC [23]. Treating xenograft-carrying mice with a demethylating agent and then screening the xenografts for altered gene expression would provide a more cancer specific discovery approach which could potentially increase the yield of novel and truly cancer-related aberrantly methylated genes identified by the screen. Although our results indicate that cell line methylation is not representative of primary tumors, cell lines are still useful for demonstrating the biological effects of hyper- and hypomethylation. Cell lines still provide a useful platform for conducting functional experiments on genes affected by altered methylation in cancer, such as gene knock-down or gene over-expression to validate the biological significance of aberrant methylation events in cancer. However, these data indicate that cancer cell lines are a poor representation of promoter methylation events in primary tumors, and that substantial artifact related to epigenetic alterations and promoter methylation exists as an artifact of adherent cell culture.

In addition to promoter methylation discovery, our findings also have implications for drug discovery in all human cancers. With the emergence of whole-genome SNP, genome-wide methylation, and gene expression microarrays, among other platforms, it has become possible to conduct integrative pathway analysis in any human cancer to identify the core key pathways involved in cancer development and progression [24]. Tumor xenografts are an attractive model for testing novel therapies targeting components of nodes of interest in core pathways. A number of previous studies have demonstrated that tumor xenografts grown in immune compromised mice may be useful in drug development [25], [26], [27], [28]. It has also been suggested that xenografts could have a role in personalized medicine by allowing for the *in vivo* testing of chemoresponsiveness of a patient's tumor [29], [30]. One limitation to using xenografts in personalized medicine is the low engraftment rate of primary tumors in mice, however, a recent study has shown that tumor-derived non-small cell lung cancer (NSCLC) implanted under the renal capsule of NOD-SCID mice have a 90% engraftment rate and grow rapidly enough to potentially be useful for aiding in the direction of a patient's chemotherapeutic regimen [31]. It has been previously shown that tumor xenografts have global gene expression profiles that are very similar to the primary tumors from which they were derived [32]. Our results demonstrate that tumor xenografts have methylation signatures that are identical to the primary tumors.

These data demonstrate that xenografts have a global methylation pattern that is highly representative of the primary tumor from which they are derived and that tumor-derived cell lines have a distinctly different methylation signature than both primary tumors and tumor xenografts. Although we did not investigate methylation difference between primary tumors, xenografts and cell lines in other solid tumors it is likely that the differences seen here, which we believe are attributable to cells being grown in culture, will also be seen in cancer cell lines derived from other human solid tumors.

## Supporting Information

Table S1 **Minimally transformed cell lines vs cancer cell lines.** Illumina probe ID and Gene name are listed.
(XLS)

Table S2 **Normal Tissue vs Minimally Transformed Cell Lines.** Illumina probe ID and Gene name are listed.
(XLS)

Table S3 **Normal Mucosa vs Cancer Cell Lines.** Illumina probe ID and Gene name are listed.
(XLS)

Table S4 **Primary Tumors vs Cancer Cell Lines.** Illumina probe ID and Gene name are listed.
(XLS)

Table S5 **Primary Tumors vs Normal Mucosa.** Illumina probe ID and Gene name are listed.
(XLS)

Table S6 **Gene Set Analysis.** Data for minimally transformed cell lines vs. cancer cell lines, primary tumor vs cancer cell lines and primary tumor vs mucosa are provided.
(XLS)

Table S7 **Gene list used for selection of bisulfate sequencing primer targets.** Illumina probe ID, gene name, and statistical significance of each target is provided.
(XLS)

Table S8 **Genes with differential methylation between Primary Tumors and Cancer Cell lines in this study and in Houshdaran et. al., 2010.** Gene ID's are provided.
(XLS)
